# Using registry data to characterize the incidence and causes of blindness in Oregon

**DOI:** 10.1371/journal.pone.0220983

**Published:** 2019-08-08

**Authors:** Mitchell V. Brinks, Travis Redd, William E. Lambert, Tosha Zaback, Joan Randall, Teresa Field, David Wilson

**Affiliations:** 1 Casey Eye Institute, Oregon Health & Science University, Portland, OR, United States of America; 2 School of Public Health, Oregon Health & Science University, Portland, OR, United States of America; 3 Oregon Commission for the Blind, Portland, OR, United States of America; Medizinische Universitat Graz, AUSTRIA

## Abstract

In the United States, there is no reliable data to describe the prevalence of eye diseases leading to visual impairment and little active surveillance to address this knowledge gap. Data that is readily available from many state blind registries may provide helpful information on trends and causes of blindness. We analyzed new registrations with the Oregon Commission for the Blind (OCB) and Oregon State Department of Administrative Services (DAS) from 1961 to 2016 for causes of and trends in blindness. Persons with blindness self-refer into the OCB registry and the Oregon State Department of Administrative Services (DAS) includes those receiving social security disability financial support and other state services. Data for 9,273 blind persons registered were analyzed. The most frequent causes of blindness were age related macular degeneration (AMD) 3,308 (38%), followed by diabetic retinopathy (DR) 729 (8%), congenital conditions 697 (8%), optic nerve atrophy 611 (7%), glaucoma 549 (6%), retinitis pigmentosa 546 (6%), retinopathy of prematurity192 (2%), cataract 180 (2%), and trauma 174 (2%). The mean age of onset of blindness was younger for Blacks (31 years) and Hispanics (33 years) than for Whites (44 years). Analysis of state-based registries can provide useful and locally relevant vision and eye health data where little information is otherwise available.

## Introduction

Reliable estimates of population burden of visual impairment and blindness are generally not available at the level of states or regions, hindering efforts for health services planning. The need to improve surveillance of visual impairment has been recognized by the National Academies of Science and Engineering [1], yet until active surveillance systems are established, other resources must be utilized. Population vision and eye health is expected to vary on urban and rural scales, and to be affected by demographic and socioeconomic factors, and local health care resources and policies. Data from individual epidemiologic studies, local health agencies and hospital service sources are likely not able to be representative at the level of an entire state or characterize time trends.

To assess our statewide trends and causes of legal blindness, we utilized registrant data from the Oregon registry sources collected during 1961 to 2016. Our purpose was to contribute to the evidence base to inform the design of an active surveillance system and public health programs. In this report, we describe our methods for use of registrant data, and findings for Oregon. Given that many states have similar service agencies and data, our approach may easily be replicated. And because blindness is the ultimate result of many eye diseases, its epidemiologic characterization in a state’s population helps to inform the design of programs for prevention, screening, treatment, and disability.

## Materials and methods

This study was approved by the Institutional Review Board of OHSU and adheres to the guidelines of the Declaration of Helsinki.

### Data source

The Oregon Commission for the Blind (OCB) is a state agency that provides rehabilitation services to residents who experience vision loss and need specialized training and support to live full and productive lives. Each month the OCB receives a list from the Oregon State Department of Administrative Services (DAS) of newly declared persons with blindness generated by interaction between these individuals and various state agencies including applications for disability insurance, referrals from health care agencies, Veterans Administration, Oregon Vocational Rehabilitation Services, and reports from the Department of Motor Vehicles as potential OCB registrants. The OCB then reviews this list and identifies individuals that are current or former clients of OCB or are already on the registry. Those who are not already on the registry are incorporated into the registry. In addition, individuals can also contact the OCB independently for services.

To be included in the OCB registry, an individual must be legally blind in accordance with U.S. law, which is having a best corrected visual acuity in the better seeing eye of equal to or worse than 20/200 or a visual field of less than 20 degrees. An ophthalmologist confirms legal blind status and determines cause and date of onset through clinical chart reviews or additional examination. An OCB staff member enters information from the ophthalmologist into the database. Demographic information is self-reported. All documentation from the OCB on-staff Ophthalmologist is maintained for internal and external audit purposes. The OCB operates with both state and federal funding therefore undergoes regular audits of these processes. The authors were provided the complete dataset for the requested time period.

### Study design

This is a retrospective analysis utilizing an existing administrative registry of new cases of blindness during the years 1961 through 2016. The OCB provided de-duplicated and de-identified data which included date of registration, age, sex, race, ethnicity, county of residence, and cause of blindness. Estimates for the date of onset of blindness were determined by an ophthalmologist conducting chart reviews of previous ophthalmology or optometry examinations. The number of OCB registrants at any one time was not available, so estimates of prevalence of blindness were not feasible. This database has not been used previously for scientific research. Data on the size of Oregon’s population was obtained from the U.S. Census [2, 3].

### Data analysis

Differences in proportions of causes of blindness among gender and racial/ethnic groups were tested using chi-square. Average annual incidence of blindness registration rates per decade were calculated using registry counts in the numerator and U.S. Census data for Oregon in the denominator. Because the state population represents an open cohort, the average population for each inter-decadal period (beginning–end) / 2) was used in the denominator. Trends in the mean age of registration were modeled on age categories using other national population eye studies which assess those over 40 years of age and analyzed using ANOVA and multivariate linear regression. Excel 2011 (Microsoft, Redmond, WA) was used for data management, and statistical analysis was performed using Stata MP 13 (StataCorp, College Station, TX).

## Results

From 1961 through 2016, a total of 9,273 persons with legal blindness were registered by the OCB and DAS. Most were White (85%) and over 40 years old (79%), and the leading cause of blindness was AMD (35%) (Table 1).

**Table 1:** Causes of blindness by age, racial/ethnic, and gender groups, Oregon Commission for the Blind data, 2007–16.

Cause	Age groups n (col %) ^a^		Race/Ethnicity n (col %) ^b^						Gender n (col %)		Total n (col %) ^a^
	< 40 yrs	≥ 40 yrs	White	Black	Hispanic	Asian	Pacific Islander	AI/AN ^b^	Male	Female	
AMD ^d^	10 (2)	897 (41)	694 (35)	7 (11)	8 (6)	5 (10)	2 (10)	18 (26)	724 (44)	283 (23)	907 (35)
DR ^e^	31 (6)	165 (8)	135 (7)	6 (9)	18 (12)	6 (12)	5 (24)	9 (13)	116 (7)	100 (8)	196 (7)
Congenital conditions	143 (25)	76 (4)	152 (8)	6 (9)	23 (16)	10 (19)	2 (10)	9 (13)	110 (7)	126 (10)	219 (8)
Optic nerve atrophy	67 (12)	93 (4)	128 (6)	6 (9)	11 (8)	5 (10)	1 (5)	4 (6)	76 (5)	99 (8)	160 (6)
Glaucoma	15 (3)	177 (8)	134 (7)	17 (26)	11 (8)	8 (15)	0 (0)	7 (10)	98 (6)	106 (9)	192 (7)
Retinitis pigmentosa	61 (11)	81 (4)	115 (6)	6 (9)	12 (8)	3 (6)	3 (14)	4 (6)	70 (4)	85 (7)	142 (5)
OCRetinal diseases/ injuries	17 (3)	100 (5)	92 (5)	3 (5)	10 (7)	3 (6)	0 (0)	3 (4)	66 (4)	63 (5)	117 (4)
ROP ^f^	47 (8)	8 (0)	46 (2)	0 (0)	3 (2)	2 (4)	0 (0)	2 (3)	27 (2)	33 (3)	55 (2)
Cataract	9 (2)	6 (0)	15 (1)	0 (0)	1 (1)	0 (0)	0 (0)	1 (1)	7 (0)	9 (1)	15 (1)
Trauma	26 (5)	23 (1)	33 (2)	3 (5)	5 (4)	2 (4)	1 (5)	0 (0)	9 (1)	47 (4)	49 (2)
Corneal/scleral conditions	4 (1)	16 (1)	12 (1)	1 (2)	3 (2)	0 (0)	1 (5)	1 (1)	13 (1)	8 (1)	20 (1)
Myopia	6 (1)	19 (1)	18 (1)	1 (2)	1 (1)	0 (0)	0 (0)	0 (0)	12 (1)	13 (1)	25 (1)
Other^g^	133 (23)	523 (24)	398 (20)	13 (20)	39 (27)	8 (15)	6 (29)	12 (17)	338 (20)	257 (21)	656 (24)
Total n [row %] ^a^	569 [21]	2,184 [79]	1,972 [85]	69 [3]	145 [6]	52 [2]	21 [1]	70 [3]	1666 [58]	1229 [43]	2753

^a^ Column and row percentages may not sum precisely to 100 due to rounding

^b^ Race/Ethnicity data missing for 15% of registrants

^c^ AI/AN = American Indian / Alaska Native

^d^ AMD = age-related macular degeneration

^e^ DR = diabetic retinopathy

^f^ ROP = retinopathy of prematurity

^g^ Other = (unknown, multiple syndromes, multiple eye conditions, achromatopsia, albinism, amblyopia, aniridia, aphakia, colomboma, cone-rod dystrophy, congenital eye defects, cortical visual impairment, hemianopia, keratoconus, leber’s congenital amaurosis, microophthamia, nystagmus, optic nerve hypoplasia, retinal detachment, retinoblastoma, stargardt’s disease, strabismus, usher syndrome)

**Fig 1:**
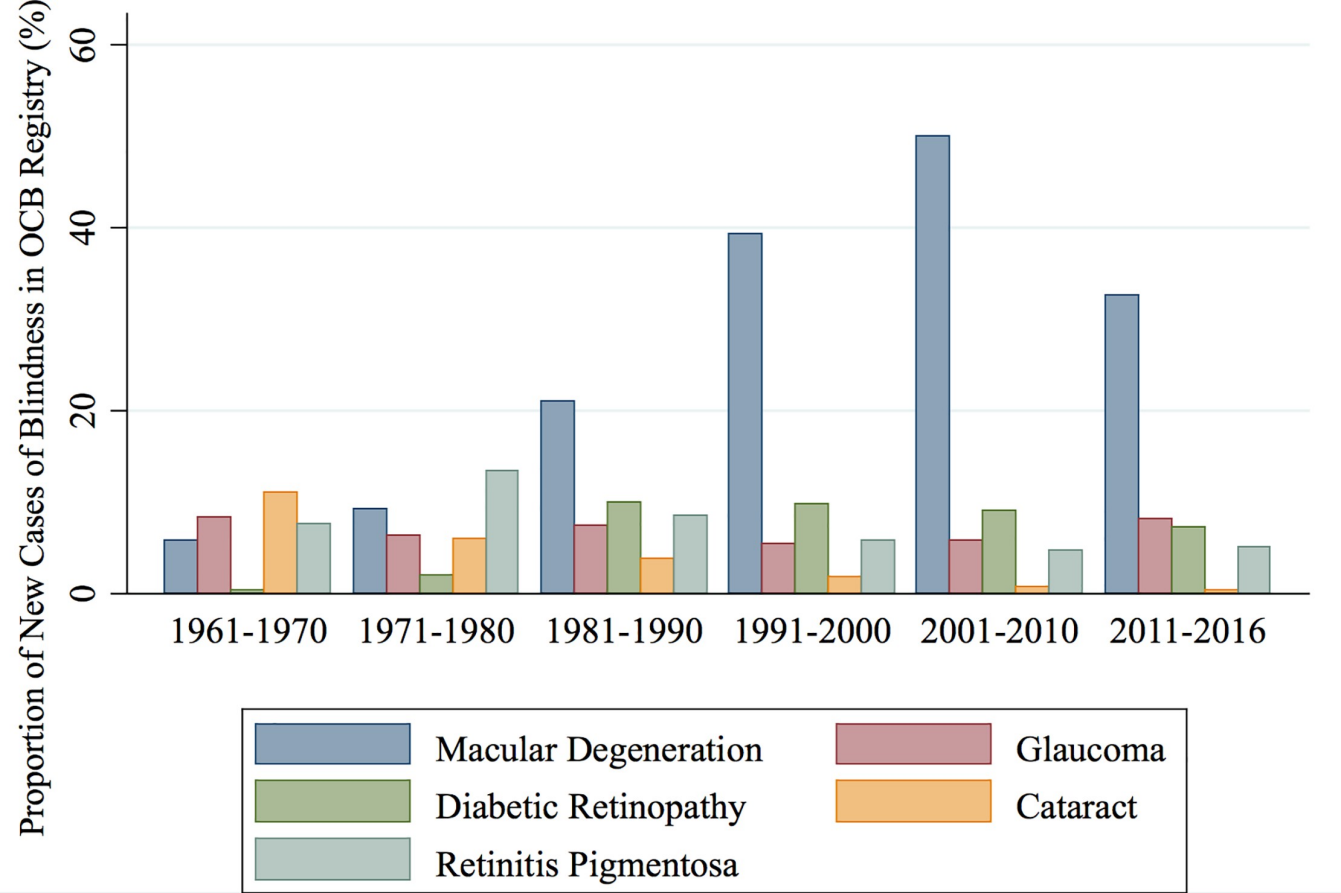
Major causes of blindness among new OCB registrants, 1961–2016.

The role of several leading causes of blindness changed from 1961 to 2016 (Fig 1). Cataract became a much less common cause of blindness, dropping from 10% (1961–1970) to <1% (2007–2016); whereas conditions associated with diabetes (DR increased from 1% in 1961–1970 to 7% in 2007–2016), and conditions associated with an aging demographic (AMD increased from 6% in 1961–1970 to 33% in 2007–2016).

**Fig 2:**
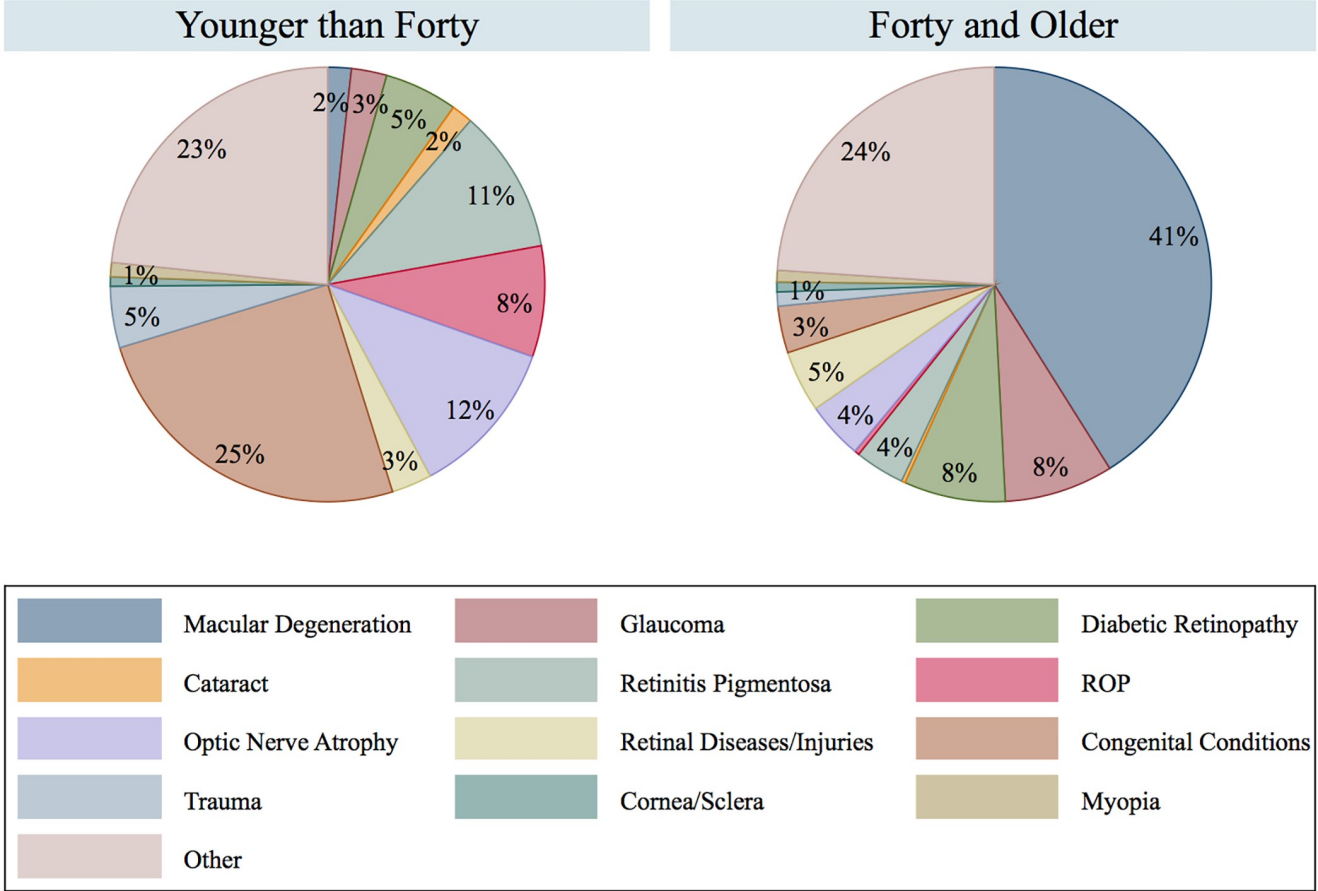
Causes of blindness by age group, 2007–2016.

For the most recent decade where data was available (2007–2016) (Table 1), the main overall causes of blindness were: AMD (35%), congenital conditions (8%), DR (7%), and glaucoma (7%). The three widely recognized contributors to the population burden of visual impairment (AMD, Glaucoma, and DR) caused more than half of blindness among those over 40 years old (total = 57%) (Fig 2). Under 40 year old OCB registrants more often lost vision from congenital conditions, optic nerve problems, retinitis pigmentosa, and ROP (total = 56%). In males, all ages combined, the three most common causes of blindness were AMD, DR, and glaucoma, as compared to AMD, congenital conditions, and DR in females, all ages combined.

We also analyzed these data for the age of onset for blindness. For the years 2007–2016, the mean age of OCB registration was 64 years (SD = 27 years) with a median of 71 years. As would be expected, the mean age at registration differed depending on the cause of blindness. The mean age of onset of blindness was 68 years for AMD, 48 years for glaucoma, 42 years for diabetic retinopathy, 29 years for trauma, and 27 years for retinitis pigmentosa (one-way ANOVA, p<0.0001). Excluding those registrants whose blindness was attributed to congenital conditions, the mean annual age of registration increased by an average of 0.8 years with each successive calendar year from 1961 to 2016 (bivariate linear regression, excluding congenital conditions, SE = 0.04 years, p < 0.0001).

**Fig 3:**
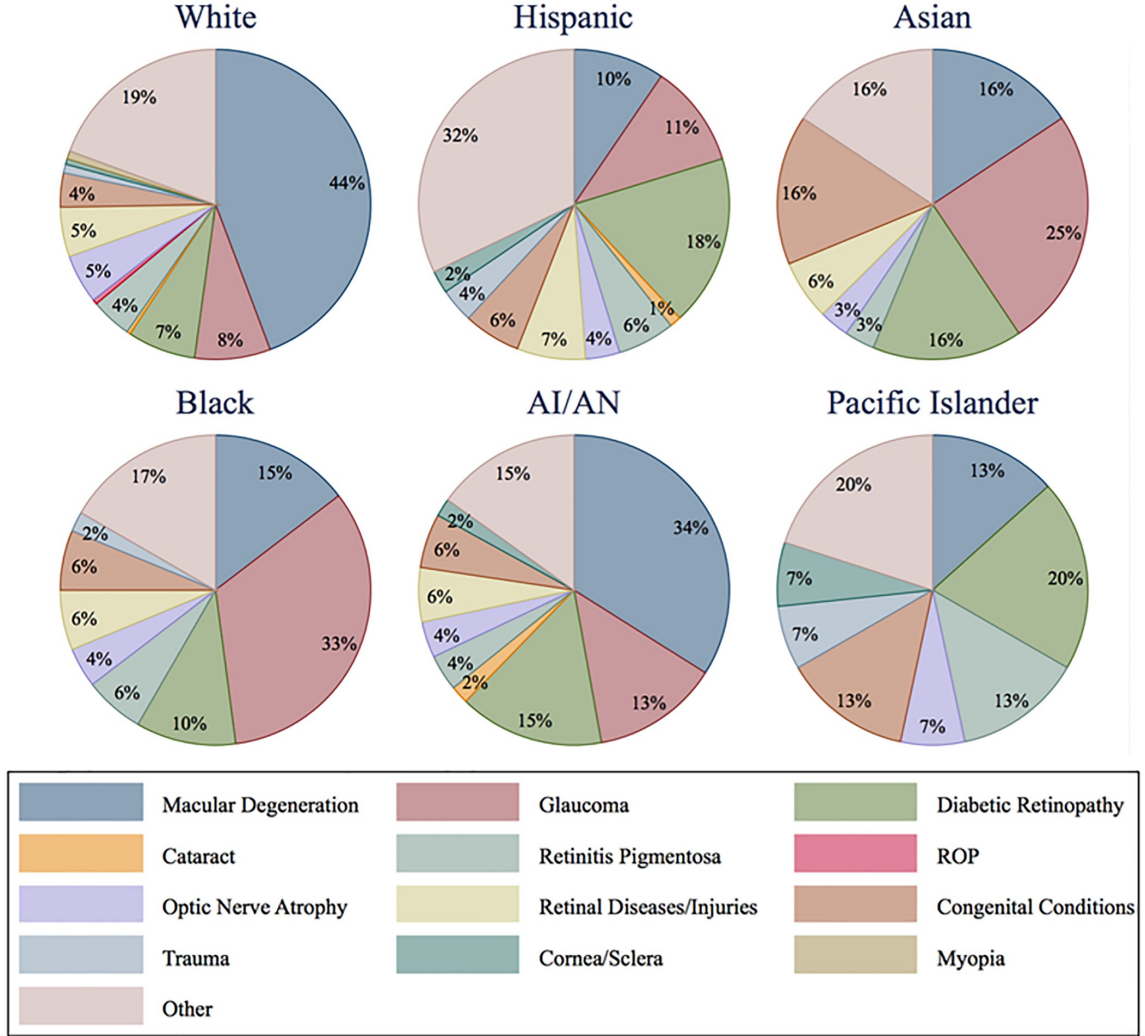
Causes of blindness over forty years, by race and ethnicity, 2007–2016.

Racial and ethnic groups demonstrated distinct patterns of blindness, for both cause and age of onset. The causes of blindness for each racial and ethnic group were analyzed among persons 40 years and older for the years 2007–16. For those 40 years and older from 2007–2016, AMD was a common cause of blindness in all groups, especially among Whites and American Indian/Alaska Natives. Among Blacks and Asians, glaucoma was the most common cause of blindness, while in Hispanics and Pacific Islanders, diabetic retinopathy was the leading cause (Fig 3). A younger mean age of onset for blindness was noted for Blacks and Hispanics compared with Whites for the entire study period and for the most recent ten-year period: Blacks (31 years, 1961–2016 vs 49 years, 2007–16) and Hispanics (33 years, 1961–2016 vs 40 years, 2007–16) when compared with Whites, (45 years, 1961–2016 vs 64 years, 2007–16). The earlier onset of glaucoma and DR blindness (e.g. vs. AMD), likely contributed to the earlier onset of blindness among Blacks and Hispanics, who frequently lost vison from these diseases.

Finally, data was analyzed by causes of disease from 2007–2016 for three categories generally representative of children (< 18 years), working age adults (19–64 years), and older adults (65 years and older) For those < 18 years of age the common causes of blindness were other (68%), congenital conditions (17%), and retinitis pigmentosa, glaucoma, cataract, and macular degenerations (each 3%). For working age adults; the common causes of blindness were other (37%), congential conditions (25%), diabetic retinopathy (16%), retinitis pigmentosa (12%), glaucoma (6%), and macular degeneration (3%). The common cause of blindness for older adults was macular degeneration (71%), congenital conditions (10%), glaucoma (10%), diabetic retinopathy (4%), and other (4%).

## Discussion

We used registry data to investigate the causes and trends of blindness among population groups in Oregon. The analysis of these established data allowed for efficient identification of subgroups at risk, diseases of particular concern, and temporal trends. This approach may be especially helpful for blindness prevention efforts given the lack of active surveillance programs and the limitations inherent to application of older estimates of general U.S. population data to unique state or regional populations. [4, 5]

Trends observed in OCB registration appear to correspond with broad population health patterns and advances in ophthalmology. The trend of increasing DR-related blindness follows a rise in the national prevalence of systemic diabetes (from 1.05% in 1961 to 7.4% in 2016). [6] The proportion of new cases of blindness attributable to DR increased by 0.3% per year from 1961 to 2000 then decreased by 0.2% per year from 2001 to 2016. The difference in these trends was not statistically significant (p = 0.42). Cataract blindness dropped from causing 10% in the 1960’s to 1% in the most recent decade, along with advances cataract surgery. Blindness due to AMD increased from 1961 through 2000 then decreased from 58.5% in 2000, to 22.2% in 2016, a trend identified in Europe and Israel as well. [7–9] The proportion of new cases of blindness attributable to AMD increased on average by 1.5% per year from 1961 to 2000 then decreased by 2.0% per year from 2001 to 2016. The difference in these trends was statistically significant (p<0.001). Recent powerful advances in diagnostic and treatment options have been credited as a major source of this downward trend for AMD.[10, 11] A marked stability characterized blindness where the benefit of clinical advance remains limited, with blindness in the per decade range of 5%-7% for retinitis pigmentosa and 7–9% for congenital conditions over the past thirty years.

Racial and ethnic patterns of vision loss revealed that the majority of blindness for Whites was due to AMD, for Blacks and Asians from glaucoma, and for Hispanics, Native Americans, and Pacific Islanders DR was especially common. These findings correspond with evidence from several large epidemiologic investigations that identified a higher risk for blindness from AMD among Whites, glaucoma among Blacks, and DR among Hispanics. [12–15] The younger onset of blindness noted among Blacks and Hispanics may add to health disparities among these groups.

One at-risk group for blindness identified by these data are Hispanics in Oregon with diabetes. While the prevalence of systemic diabetes was 15.4% in Hispanic and 21.9% in Black populations, [16] 21% of Hispanics versus 9% of Blacks 40 years and older experienced blindness from DR. This may reflect the impact of geographic barriers to care as many of Oregon’s Hispanics reside in rural areas where there are few ophthalmologists. [17] In contrast, most Blacks in Oregon reside in the state’s largest urban area near the highest density of state eye care resources and lost vision most frequently from glaucoma. This juxtaposition suggests the potential value of programs to improve access and awareness efforts among specific population groups. [3]

## Limitations

Health registry data such as that from the OCB and DAS may include biases, particularly due to participation bias which we are unable to quantify. Low socioeconomic status may increase barriers to services or increase financial incentives to register. Racial or ethnic group participation influences include distrust of government, more common among Native Americans and rural residents.[18] Potential for greater scrutiny of immigration status may preferentially discourage some groups. Each cause of blindness may also introduce bias, as diseases like diabetes may encourage greater integration into structured health care, where older patients with disease isolated to the eye, such as AMD, may be less facile with health care systems and state services.

Compared to Oregon’s statewide demographics, a modest underrepresentation in the OCB was noted for females (43% of OCB registrants versus 50.4% for Oregon) and close approximation for Whites (85% versus 87.1%) (Table 1).[19] Blindness projections from older population based studies suggest that, unlike in the OCB data, females with blindness due to AMD should outnumber men. This observation may derive from lower interest in disability support among older white females, who may have been less likely to be wage earners.

**Fig 4:**
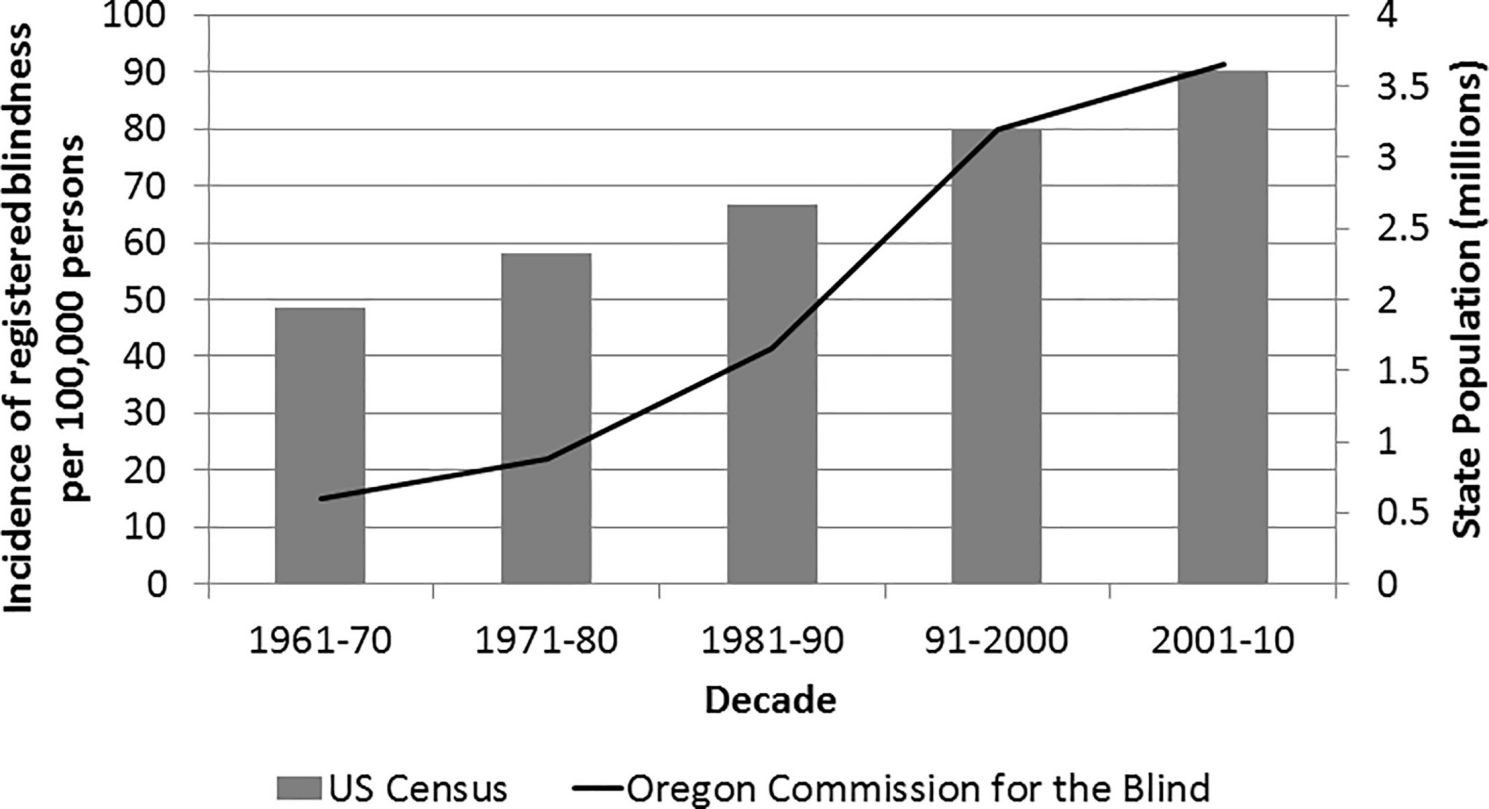
Incidence of registered persons with blindness and total state population by decade, 1961–2010.

The inclusion of state disability registration in these data likely increase data capture for those less interested in OCB services, yet motivated by social security disability insurance payments and other support. As well, blind commission administrators previously estimated 85% to 90% participation rates with these registries.[20] The plateauing of incidence in the later years of registry data (Fig 4) suggests a practical limit is now being reached in capture.

## Conclusions

Analysis of blind commission registry data provides helpful information for blindness prevention and support efforts, despite the inherent limitations of these data. Combined analysis of data from the widely distributed network of state blind commissions could efficiently add to our understanding of vision health in the U.S.
